# Positive-word stimuli via a smartphone application have no immediate-term effects on multi-directional reach ability in standing position: a randomized controlled trial

**DOI:** 10.1080/07853890.2021.1968483

**Published:** 2021-08-20

**Authors:** Kenta Azukizawa, Kodai Hirose, Yuta Morigami, Naoki Higashi, Hiroyuki Uchida, Kazuki Hirao

**Affiliations:** a Department of Rehabilitation, Oda Municipal Hospital, Oda, Japan; b Department of Rehabilitation, Ochiai Hospital, Maniwa, Japan; c Department of Occupational Therapy, Kibi International University, Takahashi, Japan; d Department of Rehabilitation, Kurashiki Heisei Hospital, Kurashiki, Japan; e Graduate School of Health Sciences, Gunma University, Maebashi, Japan

**Keywords:** Accidental falls, mobile applications, postural balance, psychological feedback, smartphone

## Abstract

**Objectives:**

The purpose of the study was to examine the immediate-term effect of positive-word stimuli via a smartphone application on the multi-directional reach ability in standing position in young adults.

**Methods:**

This study was an immediate-term, assessor-blinded, two-arm, parallel-group, randomized controlled trial among young adults recruited from one university in Japan. Participants were randomly assigned to the experimental group or control group using a computer-based random number-generating programme. Participants of the experimental group used an application on iPhone and watched 3-min videos displaying positive-word stimuli. This application repeatedly displayed positive-word stimuli every 5 s. The participants of the control group used an application on iPhone and watched the same videos as in the experimental group. However, a positive-word stimulus did not appear in the videos. The primary outcome was the multi-directional reach test (MDRT) from baseline to immediately after the intervention protocol.

**Results:**

Among the 62 randomized participants (experimental group, *n* = 31; control group, *n* = 31), 62 (100%) completed the MDRT immediately after the intervention protocol. There were no differences in mean group change values in MDRT between the experimental and control groups.

**Conclusions:**

Among young adults, positive-word stimuli via a smartphone application did not significantly improve multi-directional reach ability in standing position. These findings do not support the superiority of this intervention among young adults. **Trial Registration:** Clinicaltrials.gov, NCT03546218. Registered 6 June 2018, https://clinicaltrials.gov/ct2/show/NCT03546218

**KEY MESSAGES**
- In our study, among young adults, positive-word stimuli via an SPSRS application did not significantly improve the multi-directional reach ability in the standing position.
- These findings do not support the superiority of this intervention among young adults.

## Introduction

According to the World Health Organisation, falls are the second leading cause of accidental death or unintentional injury worldwide [1]. Every year, 37.3 million severe falls that require doctors’ consultation occur [1]. Falls are common in elderly people, and approximately one-third of them have experienced falls [2,3]. Falls are a public health problem; even if it does not lead to death, it can cause reduced physical function, limited participation and activity, social isolation, reduced confidence, and reduced quality of life, such as from fractures or head trauma [4–13]. Therefore, an effective prevention strategy against falls is important. Previous studies have suggested that a reduction in the reach ability in a standing position is a risk factor for falls [14–19]. Therefore, interventions focussing on reach ability in standing position are necessary to prevent falls.

The positive-word stimuli method using video can be cited as an intervention method to improve reachability in standing position [20]. This intervention does not require manpower and allows uniform intervention regardless of the experience of the therapist [20]. Aoyama et al. reported that the forward reach ability in standing position was improved by videos on positive-word stimuli displayed on a personal computer (PC) in young adults [20]. Therefore, positive-word stimuli using videos may contribute to the prevention of falls in elderly people. On this basis, we developed a smartphone application, Application Program Status Register (SPSRS), for greater accessibility based on research on positive-word stimuli using this video [21]. This application is free. Similarly, YouTube posts various kinds of videos that users can watch without getting bored. In addition, the positive-word stimulus is programmed to be automatically presented in the video [21]. This application is an easy-to-use intervention method, has few restrictions on place and time, and can be used for pockets of time [21].

However, the positive-word stimuli using the SPSRS application and videos on PC varied on screen size. Therefore, whether SPSRS application intervention improves forward reach ability in a standing position is unknown. In addition, Cummings et al. suggested that falls occur not only in the forward direction but also towards the side and back [22]. For this reason, Newton developed the multi-directional reach test (MDRT), a useful assessment tool that provides a simple and inexpensive way to evaluate reach ability and fall risk in the sideways and backward directions [23]. Despite the development of these sides and backward fall hazard assessment tools, it is unclear whether the SPSRS application will improve the ability to reach sideways and backward.

The purpose of this study is to clarify whether positive-word stimuli using video by SPSRS application improves forward, backward, and side reach abilities. This study was conducted for young adults as a preliminary stage of implementing SPSRS application intervention for the elderly. Moreover, this study hypothesized that the group of young adults who received positive-word stimuli via the SPSRS application has improved reach ability in the forward, side, and backward directions compared with the group who did not receive positive-word stimuli.

## Methods

### Design

This study was reported according to the CONSORT statement [24]; it is an assessor-blinded, two-armed, parallel-group, randomized controlled trial (RCT). The allocation ratio was randomly assigned in a 1:1 ratio to either the experimental group or the control group. The study was conducted with approval by the Ethics Committee of Kibi International University (#18-16) and was registered in advance at ClinicalTrials.gov (Identifier: NCT03546218). Written consent was obtained from all participants.

### Participants

Participants were recruited in Kibi International University, Takahashi, Okayama, Japan, in June 2018. Inclusion criteria were young adults aged 18–24 years. Both males and females were included. Exclusion criteria were individuals with a physical disability that can sufficiently interfere with daily life. Participants were asked to fill out a questionnaire on their age, sex, height, weight, body mass index, and exercise habits (presence or absence) before intervention. Aoyama et al. has reported that the average (standard deviation, SD) of the forward reach test in the experimental group was 32.30 (4.74) cm and that in the control group was 36.71 (4.87) cm [20]. Therefore, the difference between the minimum of the two groups we want to detect for intervention is 4.41 cm and the mean of the SD is 4.81. Therefore, to achieve 90% power at a 5% significance level on both sides, at least 52 participants in total (26 or more in each group) were required. Participants were assigned to the experimental or control group using a pseudorandom number generator and permuted block algorithm implemented in Microsoft Excel by a third party. The nature of group assignment and intervention did not allow masking of participants. Baseline and post-intervention evaluations were performed by outcome assessors who were not involved in the intervention. The outcome assessors were not informed of the group assignment of the participants throughout the trial. Thus, blinding was successful because the group assignment was not known to the outcome assessors until the trial was over. Intervention practitioners were not blinded. However, they were not involved in randomization, group assignment, data collection, or statistical analysis.

### Outcome measures

**Figure 1.**
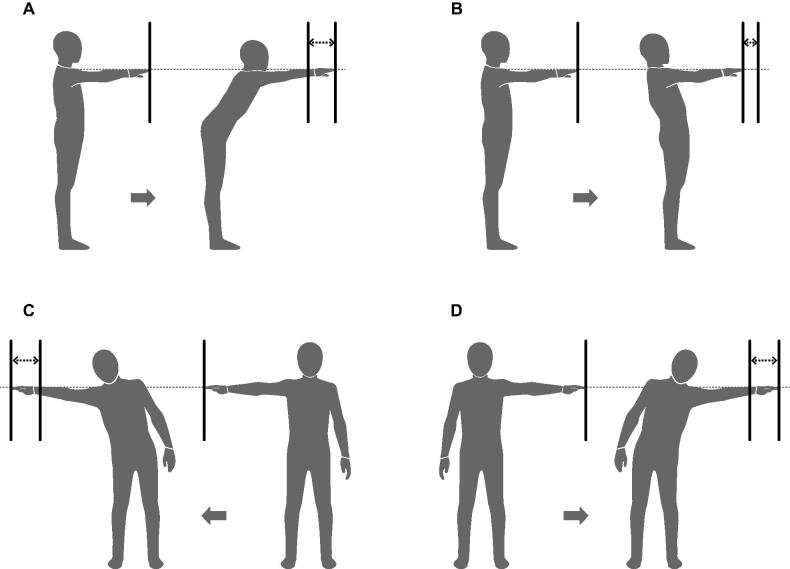
Overview of measurement method of multi-directional reach test. A. Forward reach test. B. Backward reach test. C. Right reach test. D. Left reach test.

Outcomes were measured at baseline and immediately after the intervention protocol. The primary outcome was the MDRT [23]. MDRT is a tool for measuring the reach ability and limits of stability in four directions (forward, backward, right, and left reach tests) in a standing position (Figure 1). Newton’s study has established the reliability and validity of MDRT [23]. The MDRT was measured using the GB-200 (OG Giken, Okayama, Japan). Participants wore their own shoes while they performed the MDRT, and the GB-200 yardstick was matched to the participants’ acromion process level. The procedure was measured in the order of forward, backward, right, and left directions. In the forward and backward reach tests, the participants moved their hands forward or backward as far as possible with the arm outstretched and flexed at 90° in a standing position (Figure 1). In the right and left reach tests, the participants moved their hands right or left as far as possible with the arm outstretched and abducted at 90° in standing position (Figure 1). In all tests, the sole was not allowed to leave the floor during the test. The primary measurement was the difference between the first and last fingertip positions (in cm). Each participant repeated the MDRT twice in each direction, and the best performance was used for analysis. Dynamic or static balance tests other than the MDRT were not performed in this study because we thought it was necessary to consider the duration of the intervention effect. Ruch et al. suggested that in the case of immediate intervention, the effects of word stimulation fades over time [25]. In addition, the MDRT consisted of four reach tests, and we predict that it would take about 5 min to complete these tests [26]. Given the limited effect of word stimulation on the immediate intervention and duration of the MDRT, it is possible that not all balance tests accurately measured the immediate effect of word stimulation when multiple balance tests were administered. For these reasons, we did not employ dynamic or static balance tests other than the MDRT.

### Interventions

**Figure 2.**
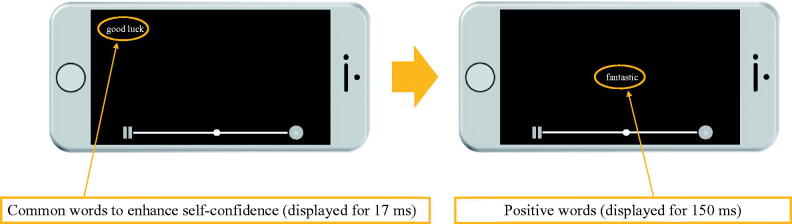
SPSRS application.

Participants were seated one by one in a chair in front of a desk in a quiet room. Both groups used the same iPhone 6 s and watched the same video (playing basketball video) for 3 min. The experimental group used SPSRS [21] as a video playback application. The control group used YouTube. The smartphone application SPSRS used in the experimental group was available in Japanese and free for iOS 9.0 and higher smartphones [21]. SPSRS can use keywords as in general video playback applications to search and watch the video. SPSRS is programmed to display common words to enhance self-confidence, such as “can”, “let us try”, “good luck”, “able”, and “do not worry” [27], and are randomly displayed at the four corners of the screen (for 17 ms each). Thereafter, positive words such as “nice”, “great”, “fantastic”, “satisfactory”, and “enjoyable” are displayed [28]. These words are displayed at the centre of the screen (for 150 ms each). These words (common words to enhance self-confidence and positive words) are repeatedly displayed every 5 s (see Figure 2). Control intervention used YouTube as the video playback application. Participants watched the same videos as the experimental group. However, the stimulus of common words to enhance self-confidence and the stimulus of positive words does not appear in the video.

### Statistical analysis

The effect of SPSRS intervention on primary outcome measurements was evaluated and analyzed using linear mixed models (LMM) with a restricted maximum-likelihood estimation method for repeated measurement analysis [29–31]. LMMs do not assume sphericity for repeated measures, as required by traditional statistical analysis methods (e.g. ANOVA), thus reducing type I error and overestimation of results [29]. In addition, compared to traditional data analysis methods, LMMs are more sensitive, making them particularly effective methods for studies with small to moderate sample sizes [29]. LMM applied the intention-to-treat principle and included all participants who provided baseline data in the analysis. For each model, the random effect was the participant, and the fixed effect was the group (experimental or control group), time, and group × time interaction. The most important analysis was to examine the difference in the mean change between the experimental group and control group from baseline to immediately after the intervention protocol as the interaction of the fixed effects between group and time. We used type III fixed effects, and the statistical significance of the *p*-value was set to less than .05. These data were analyzed using SPSS version 25.0 for Windows (IBM, Armonk, NY). The between-group effect size was calculated for the mean change value (Hedge’s *g*) [32,33]. Hedge’s *g* was calculated using the following formula [32,33]:

$$\text{Hedge's }g=\frac{{\text{Mean change}}_{E}-{\text{Mean change}}_{C}}{{sd}_{\mathrm{pool}}}[1-\frac{3}{4N-9}]$$

$${sd}_{\mathrm{pool}}=\sqrt{\frac{(n_{E}-1){sd}_{E}^{2}+(n_{C}-1){sd}_{C}^{2}}{N-2}}$$

Cohen’s standardized criteria were used to interpret the magnitude of the effect size [34]. According to Cohen’s standardized criteria, 0.2 can be considered small, 0.5 medium, and 0.8 large [34].

## Results

**Figure 3.**
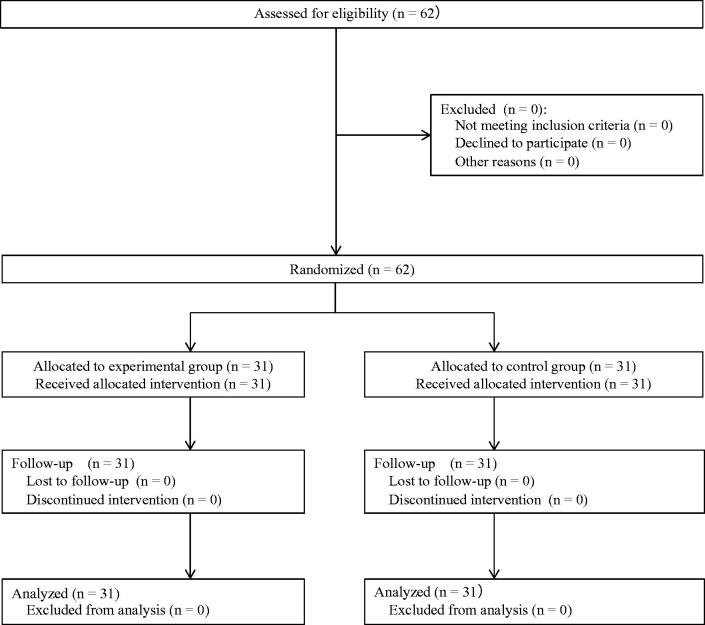
Flow of participants in the study.

In June 2018, 62 participants were assessed for eligibility. All participants met the inclusion criteria, and none of the participants were excluded. Therefore, 62 participants were randomly assigned to either the experimental group (*n* = 31) or the control group (*n* = 31). No participants dropped out during the trial. The flow diagram of the process of this study is shown in Figure 3. Table 1 shows the baseline characteristics of the two groups. Table 2 shows the estimated effect of the SPSRS application on the cited outcomes based on LMM analysis of the experimental and control groups. In addition, Table 3 displays the mean and SD of the outcome measures, as well as the effect size (Hedge’s *g*) values between the two groups. The mean forward reach test (baseline) was 31.57 cm for the experimental group and 32.03 cm for the control group. The mean forward reach test (post) was 31.21 cm for the experimental group and 31.30 cm for the control group. The mean backward reach test (baseline) was 25.04 cm for the experimental group and 24.61 cm for the control group. The mean backward reach test (post) was 26.26 cm for the experimental group and 25.58 cm for the control group. The mean right reach test (baseline) was 27.94 cm for the experimental group and 27.51 cm for the control group. The mean right reach test (post) was 28.45 cm for the experimental group and 28.26 cm for the control group. The mean left reach test (baseline) was 27.54 cm for the experimental group and 27.84 cm for the control group. The mean left reach test (post) was 28.54 cm for the experimental group and 28.61 cm for the control group. The LMM results showed no significant interaction effect between group and time for all outcome measures (*p* > .05 for all). The between-group effect size of the mean change was negligible in all outcomes (Hedge’s *g* < 0.2 for all).

**Table 1.** Baseline characteristics of the two groups.

	Experimental group	Control group
Characteristics	(*n* = 31)	(*n* = 31)
Age, years	19.39 (0.72)	19.58 (0.81)
Sex		
Male	22 (71.0%)	21 (67.7%)
Female	9 (29.0%)	10 (32.3%)
Height, cm	166.17 (9.80)	166.15 (8.76)
Weight, kg	60.26 (12.13)	62.24 (12.83)
Body mass index	21.69 (3.21)	22.46 (3.72)
Exercise habits		
Presence	14 (45.2%)	17 (54.8%)
Absence	17 (54.8%)	14 (45.2%)

Data are means (standard deviation) or numbers (%).

**Table 2.** Results of LMM analysis for the experimental and control groups.

Outcomes	*F*	df	*p*
MDRT (cm)			
Forward reach test			
Group Effect	0.048	1, 60	.828
Time Effect	1.186	1, 60	.280
Group × Time	0.135	1, 60	.715
Backward reach test			
Group Effect	0.060	1, 60	.808
Time Effect	4.102	1, 60	.047
Group × Time	0.056	1, 60	.814
Right reach test			
Group Effect	0.072	1, 60	.79
Time Effect	1.823	1, 60	.182
Group × Time	0.060	1, 60	.807
Left reach test			
Group Effect	0.024	1, 60	.878
Time Effect	4.816	1, 60	.032
Group × Time	0.076	1, 60	.784

LMM: linear mixed model; MDRT: multi-directional reach test.

**Table 3.** Results of effect size analysis between groups.

	Experimental (*n* = 31)	Control (*n* = 31)	Between group Effect size	
Variable	Mean ± SD	Mean ± SD	Hedge’s *g*	95% CI
MDRT (cm)				
Forward reach test			0.09	−0.41 to 0.59
Pre	31.57 ± 5.45	32.03 ± 5.03		
Post	31.21 ± 5.55	31.30 ± 5.19		
Backward reach test			0.06	−0.44 to 0.56
Pre	25.04 ± 7.27	24.61 ± 10.78		
Post	26.26 ± 7.68	25.58 ± 10.40		
Right reach test			−0.06	−0.56 to 0.44
Pre	27.94 ± 3.46	27.51 ± 5.65		
Post	28.45 ± 4.58	28.26 ± 5.70		
Left reach test			0.07	−0.43 to 0.57
Pre	27.54 ± 4.19	27.84 ± 5.01		
Post	28.54 ± 3.75	28.61 ± 6.34		

CI: confidence interval; MDRT: multi-directional reach test; SD: standard deviation.

## Discussion

In this RCT, positive-word stimuli using the SPSRS application did not show a difference between groups in immediate multi-directional reach ability in standing position. Specifically, a positive-word stimulus intervention using a smartphone application is not superior to an intervention that does not provide a positive-word stimulus using the application and is not beneficial as a treatment programme for evidence-based multi-directional reach ability in standing position. This result is different from Aoyama et al. in which positive-word stimulus using video improved forward reach ability [20]. Aoyama et al. randomly assigned 50 participants into a group that received subliminal priming-plus-subliminal reward stimuli in videos (experimental group: 25 participants) or a group that received subliminal priming-plus-supraliminal reward stimuli in videos (control group: 25 participants) and compared the differences in forward reach ability immediately after the intervention. Both the experimental group and the control group showed significant improvement in forward reach ability before and after the intervention. In addition, the control group showed a greater improvement (*d* = −0.92) in FRT immediately after the intervention compared to the experimental group. This discrepancy may be due to the screen size. In Aoyama et al., a positive-word stimulus was given via a PC [20]. In the present study, however, a positive-word stimulus was given via a smartphone application. In the smartphone application, the small screen may have displayed the positive word at the centre of the screen making it small and not easily recognizable to the participants. Aoyama et al. has reported that positive words are more effective when recognized by participants [20]. Furthermore, Aarts et al. and Takarada et al. have reported that grip strength improved by presenting positive-word stimulus via a PC [28,35]. This difference in screen size makes positive word recognition difficult, which can greatly contribute to the difference in results. Therefore, the positive word should be displayed at the centre of the screen in a large size so that participants can easily recognize it. In the future, a trial using larger characters displayed on the SPSRS application is warranted.

Participants in this study had higher baseline MDRT values than those in Tantisuwat et al. [36]. Moreover, in this study, the intervention effect was possibly not recognized because of the ceiling effect, as the participants had originally high reach ability in standing position. Therefore, it may be desirable for future trials to include participants with reduced reach ability in standing positions. In addition, Leirós-Rodríguez et al. showed that postural control and standing balance while walking may differ depending on age and gender [37–39]. It is necessary, therefore, to consider age and gender differences in future trials.

Defining the timing and frequency of positive-word stimuli is ongoing. A study conducted on patients with mild disabilities observed that repeated encouragement did not improve physical activity [40]. According to Dobkin et al., a reason for this observation is that verbal encouragement was not frequent enough to generate sufficient motivation to change behaviour [41]. Similarly, the timing and frequency of positive-word stimuli were possibly low in this study. Different intervention schedules may have been more effective (e.g. longer period, longer time, and more frequent). Therefore, future study should examine the timing and frequency of positive-word stimuli in detail.

This study has several strengths. For example, this is the first RCT to examine the influence of positive-word stimuli using the SPSRS application on multi-directional reachability in standing position. There were no dropouts and participants completed all assessments. The automated nature of the application intervention did not depend on the therapist’s experience and provided a uniform intervention for all participants. In addition, the application intervention has low restrictions on time and place. As a result, our application intervention is very generalizable to daily treatment programmes.

Nevertheless, this study had some limitations. Participants in this study were recruited from one university. Therefore, the study participants do not represent the entire population of young adults. Future studies may need to collect samples from multiple regions. Second, older adults were not included in this study. Therefore, the study results cannot be directly applied to this population. Finally, this study examined only the immediate effects of positive-word stimuli using an SPSRS application intervention. Therefore, the long-term effects of this intervention cannot be inferred. Future studies will be needed to examine the long-term effects of positive-word stimuli via this application.

In conclusion, among young adults, positive-word stimuli via an SPSRS application did not significantly improve the multi-directional reach ability in the standing position. These findings do not support the superiority of this intervention among young adults.

## Data Availability

The anonymized dataset is available from the corresponding author on reasonable request.
